# Light-driven locomotion of a centimeter-sized object at the air–water interface: effect of fluid resistance†

**DOI:** 10.1039/c9ra01417a

**Published:** 2019-03-13

**Authors:** Hisato Kawashima, Akihisa Shioi, Richard J. Archer, Stephen J. Ebbens, Yoshinobu Nakamura, Syuji Fujii

**Affiliations:** Division of Applied Chemistry, Graduate School of Engineering, Osaka Institute of Technology 5-16-1 Omiya, Asahi-ku Osaka 535-8585 Japan; Department of Chemical Engineering and Materials Science, Doshisha University Kyoto 610-0321 Japan ashioi@mail.doshisha.ac.jp; Department of Chemical and Biological Engineering, The University of Sheffield Mappin Street Sheffield S1 3JD UK; Department of Applied Chemistry, Faculty of Engineering, Osaka Institute of Technology 5-16-1 Omiya, Asahi-ku Osaka 535-8585 Japan syuji.fujii@oit.ac.jp; Nanomaterials Microdevices Research Center, Osaka Institute of Technology 5-16-1 Omiya, Asahi-ku Osaka 535-8585 Japan

## Abstract

A centimeter-sized flat-headed push pin with photothermal properties can be moved on a water surface by a simple near-infrared laser. Using light as an external stimulus allows for the remote control of the timing, direction and velocity of its locomotion. It has been clarified that the vertical orientation of the pin at the air–water interface affects the friction of locomotion, and therefore velocity and acceleration. The pin placed on a water surface with a pin point upward (a point protruding into air phase) moved an average distance of 5.3 ± 2.9 cm following one pulse of laser irradiation, and that placed with a pin point downward (a point protruding into water phase) moved 2.0 ± 1.4 cm. The velocity and acceleration were larger when the pin was placed on the water surface with a pin pointing upward, compared to when placed with the pin pointing downward. Numerical analysis conducted for the locomotions of the pin concluded that the differences in traveling distance, velocity and acceleration were due to the difference in fluid resistance of the pin point in air and water phases during their locomotion. This demonstration of remote control of the motion of small objects by light can open up a wide range of future transport applications.

## Abstract 2

Centimeter-sized flat-headed push pin with photothermal properties can be moved on a water surface by a simple near-infrared laser.

## Graphical abstract

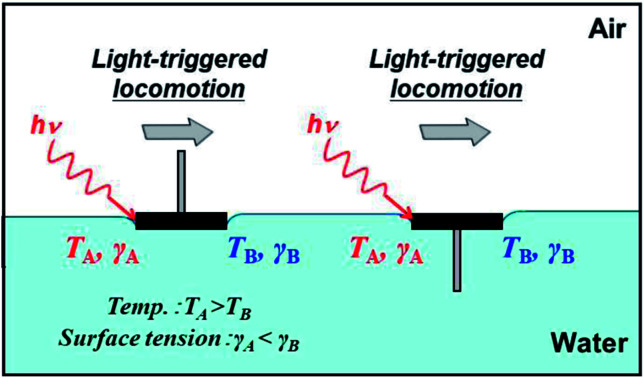

## Introduction

Powering and controlling the locomotion of micrometer- to centimeter-sized small objects is a fascinating research topic^1–4^ with possible applications in drug delivery and microfluidics. In order to power the locomotion of small objects, interfacial chemistry (*e.g.*, chemical reactions and gradients in surface tension) has been utilized. For example, generation of oxygen *via* catalytic decomposition of hydrogen peroxide is a well-known driving force to move small objects.^5–9^ Additionally, a surface tension gradient-induced Marangoni flow can lead to powerful propulsion of small objects.^10–12^ In a similar manner, soap boats,^13^ camphor crystals,^14^ organic solvent-loaded objects^15,16^ and depolymerizable plastics^17^ can move on a planar water surface due to Marangoni propulsion: the dissolution of chemicals, which decrease surface tension of water, creates a surface tension gradient at the air–water interface, which induces motion. Recently, light-induced Marangoni flow has been proven to work as a powerful propulsion force to move small objects.^18–25^ Directional light irradiation of small objects with photothermal properties converts light into heat, and the heat diffuses to the water surface giving an asymmetric thermal distribution which generates a gradient in surface tension and results in locomotion of the objects on the planar air–water interface. These studies contribute to the development of a new research area, namely, active soft matter. There has been an increasing amount of research devoted to the locomotion of small objects floating on planar liquid surfaces, however, there are few studies on the locomotion of small objects with parts protruding into the liquid phase. Considering the development of smart locomotion of small objects on liquid surfaces, investigation into the movement of complex-shaped objects carrying parts protruding into liquid phase will be crucial.

**Fig. 1:**
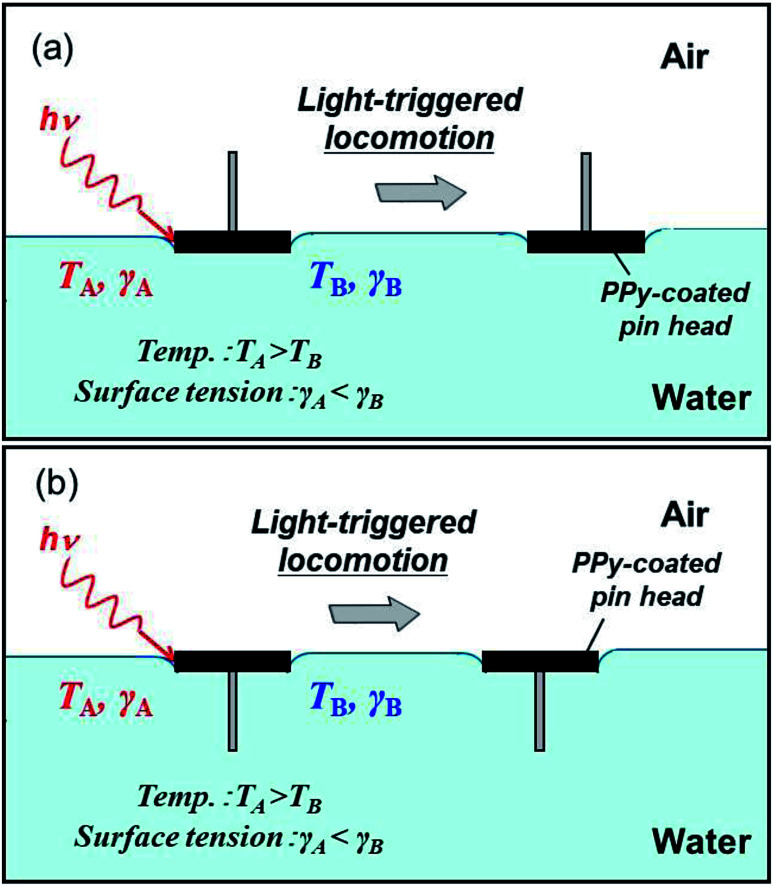
Scheme illustrating the light-driven locomotion of pin-shaped objects. The pins can be moved on the planar air–water interface with the pin points protruding into (a) air and (b) water phases. NIR laser irradiation of the pins covered with hydrophobic polypyrrole (PPy) powder converts light into heat, generating a thermal surface tension gradient. This results in locomotion of the pins on the air–water interface.

Here, we investigated the locomotion of a centimeter-sized flat-headed push pin on water surface driven by light (Fig. 1). Irradiation of the pin coated with photothermal materials caused locomotion at the air–water interface. The placement direction of the pin on an air–water interface affects the fluid resistance, and therefore velocity, acceleration and traveling distance. Being able to control the locomotion of the small objects by structural design of the objects should open up a wide field of conceivable applications.

## Experimental section

### Materials

Unless otherwise stated, all materials were guaranteed reagent grade. Ferric chloride (FeCl_3_·6H_2_O), heptadecafluorooctane sulfonic acid (C8F, 40 wt% aqueous solution), agar (ash 2.0–4.5%) and aluminium oxide (activated, basic, Brockmann 1, standard grade, ∼150 mesh, 58 Å) were obtained from Sigma-Aldrich and were used without further purification. Pyrrole (Py, 98%) was also obtained from Sigma-Aldrich and purified by passing through a column of the activated basic alumina prior to storage at −15 °C before use. Deionized water (<0.06 μS cm^−1^) was prepared using a deionized water producing apparatus (Advantec MFS RFD240NA: GA25A-0715) and used for syntheses and purification of the polypyrrole (PPy). Pin (Pushpin C.P., Tokyo, Japan) was purchased from Lemon Co., Ltd., Tokyo, Japan.

### Synthesis of hydrophobic PPy bulk powder with photothermal properties

Chemical oxidative polymerization in the presence of fluorinated dopant, C8F, was conducted to synthesize hydrophobic PPy based on the method reported elsewhere.^26^ C8F (1.0 g) was added as aqueous solution (40 wt%, 2.5 g) by a pipet to water medium (90 g) containing Py (1.0 g, Py/C8F, 3/0.4 molar ratio) in a 200 mL screw-capped bottle and the system was stirred with a magnetic stir bar for 15 min. FeCl_3_·6H_2_O oxidant (9.4 g, Py/FeCl_3_, 3/7 molar ratio) was dissolved in 10 g water and then added to the aqueous solution of Py and C8F. The polymerizations were allowed to proceed for 24 h at 23 °C and 500 rpm. The resulting PPy was black-colored and subsequently purified by repeated centrifugation–redispersion cycles (successive supernatants were replaced with deionized water) in order to remove the unwanted water soluble chemicals (free dopant and HCl). The redispersion was conducted by sonication for more than 30 min using a Bransonic C221 (Yamato, Co.). The samples were washed over ten times with deionized water, followed by freeze drying overnight.

### Characterization of hydrophobic PPy bulk powder

Morphology of the PPy bulk powder was studied using a field-emission scanning electron microscope (FESEM) (SU8020 instrument, Hitachi High-Technologies Co., Tokyo, Japan). The PPy powder placed on an aluminum stub was sputter-coated with gold using an Au coater (SC-701 Quick Coater, Elionix, Tokyo, Japan) in order to minimize sample-charging problems. X-ray photoelectron spectroscopy (XPS) analysis was conducted to characterize surface chemistry of the PPy powder using an AXIS Ultra spectrometer equipped with a monochromated AlKα X-ray gun (Kratos Analytical Ltd, Manchester, UK). For XPS analysis, the dried PPy powder sample was adhered to conducting tape, and the surface was completely covered with the powder. Static contact angles of liquid droplets (5 μL) were measured on pressed pellets 30 s after the deposition using an SImage02 apparatus (Excimer Inc., Kanagawa, Japan).

### Fabrication of small objects

**Fig. 2:**
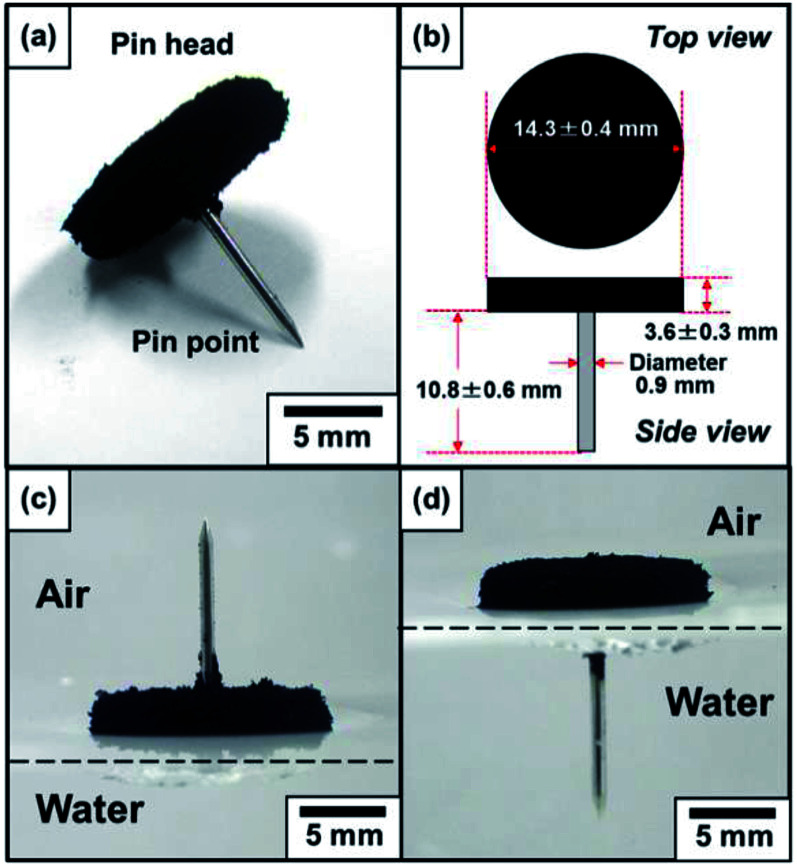
(a) Stereophotograph and (b) dimensions of a pin with the pin head coated by hydrophobic PPy powder. The pin was placed on a paper. (c and d) Stereophotographs of the pin placed on the planar air–water interface with the pin point protruding into (c) air and (d) water phases.

The centimetre-sized objects were prepared as follows: first, the pin was filed to have planar heads with a weight of 320 ± 9 mg (diameter of pin head, 13 mm; thickness of the pin head, 2.2 mm; diameter of cylindrical shaped pin tip, 0.9 mm; length of the pin tip, 11 mm), and only the head part was coated with agar gel aqueous solution at 70 °C, cooled down to room temperature to solidify the gel (agar gel layer thickness, 1.3 ± 0.3 mm; agar gel layer weight, 320 ± 460 mg; length of needle, 10 mm), and then coated with the hydrophobic PPy powder. Thanks to the PPy powder coating, the pin with hydrophobic surface can be placed on the planar air–water interface and light-to-heat photothermal property could be introduced to the pin. (Note that without any PPy coating, the hydrophilic pin went under bulk water phase.) The composite pins were partly dried under ambient conditions for 24 h, and the final weight of the composite pin was 478 ± 41 mg (diameter of head, 14.3 ± 0.4 mm; thickness of head, 3.6 ± 0.3 mm; diameter of needle, 0.9 mm; length of needle, 10.8 ± 0.6 mm), and the weight ratio of pin/agar gel/PPy powder was gravimetrically measured to be 66.4/32.4/0.3 respectively (Fig. 2a and b).

### Light-controlled movement

A near-infrared (NIR) laser (808 nm; spot diameter, 1 mm × 5 mm; output power, ∼200 mW) was utilized. The light was manually directed sideways onto the pins floating at the air–water interface (the depth of water is approximately 15 mm.) from a distance of approximately 6–8 cm, aiming for the three-phase (solid–liquid–air boundary) contact. The angle of irradiation played a crucial role. An angle of up to 45°, with respect to the plane of the water, was most efficient at propelling the pin. At 90°, *i.e.*, vertical irradiation, the pin typically remained stationary. Accurate laser positioning on the three-phase contact was difficult by hand, which caused fluctuation of the velocity and acceleration of the resulting locomotion. To simplify aiming at the pin, a NIR laser detection sheet was placed underneath the Petri dishes containing the water and acrylic glass (IR sensor card 800–1600 nm, LDT-008, Laser Components GmbH, Olching, Germany). (Note that the visible laser dot stems from the laser detection sheet which was underneath the water bath. Whenever the laser light did not hit the pin to be converted to heat it was converted to visible light by hitting the laser detection sheet. The shift in perspective of the visible laser dot and the pin results from the upper back side illumination of the pin and the height difference between the pin floating on the water bath and the laser detection sheet.) A digital video camera (Sony Handycam HDR-CX270 V; 30× optical zoom lens, Sony Co., Tokyo, Japan) was used to record movies and photographic images of the pins, and their locomotion was recorded using a digital camera (Ricoh G700SE; 5.0× optical zoom lens, Ricoh, Tokyo, Japan).

### Numerical analysis

The movies were analysed using the software Image-J to obtain the position coordinates. Velocity, acceleration and applied force were calculated from them. We first determined the position of the center of mass of the objects every frame, *r*(*t*). Next, we obtained the finite difference of the position (displacement), Δ*r*(*t*) from the time course of *r*(*t*), and the velocity *v*(*t*) = Δ*r*(*t*)/Δ*t*, where Δ*t* is the video frame rate (1/30 s). The average over 30 frames 〈*v*(*t*)〉 was calculated to reduce the noise. From the 〈*v*(*t*)〉, we obtained the acceleration *a*(*t*) = (〈*v*(*t* + Δ*t*)〉 − 〈*v*(*t*)〉)/Δ*t*. The average of the acceleration 〈*a*(*t*)〉 over 30 frames was used. The force acting on the objects *F*(*t*) is calculated from *m*〈*a*(*t*)〉, where *m* is the mass of the object. The force *F*(*t*) is equal to *F*_NIR_(*t*) + *F*_drag_(*t*), where *F*_NIR_(*t*) and *F*_drag_(*t*) represent the force caused by laser irradiation and fluid resistance, respectively. When the object was moved by its inertia without laser irradiation, *F*_NIR_(*t*) = 0. At and after *F*_NIR_(*t*) became zero, *F*_drag_(*t*) was obtained to evaluate the fluid resistance (drag) coefficient *C*_D_ that depends on the object shape and the Reynolds number (Re). Once the relationship between *C*_D_ and Re had been obtained, this relationship was used to evaluate *F*_drag_(*t*) in the laser irradiation stage. Then, *F*_NIR_(*t*) was calculated from *F*(*t*) − *F*_drag_(*t*). In the calculation for this laser irradiation stage, 〈*v*(*t*)〉 and 〈*a*(*t*)〉 were averaged over 10 frames, because the duration of laser irradiation was, at least, 1.0 s.

## Results and discussion

The centimeter-sized push pin with photothermal properties was prepared by coating the pin head with black-colored polymer, polypyrrole (PPy) (Fig. 2a and b). PPy is known to have photothermal properties,^27^ and aqueous chemical oxidative polymerization in the presence of perfluoroalkyl dopant, C8F, was conducted to synthesize hydrophobic PPy. The sulfonate group is known to be an efficient dopant anion for PPy, and has stronger interaction with PPy compared to chloride ion from FeCl_3_ oxidant.^28^ X-ray photoelectron spectroscopy studies confirmed that the surface Cl/N atomic ratio was measured to be 0.14, which was lower than that determined for PPy bulk powder synthesized using FeCl_3_·6H_2_O in the absence of C8F (0.22)^26^ (Fig. S1†). This result strongly indicated that the PPy was doped with C8F carrying sulfonate group, and therefore, it can be expected that the perfluoroalkyl group exists at the surface of the PPy. FESEM studies confirmed that the resulting dried PPy bulk powder consisted of flocs of a few hundreds nm-sized atypical PPy particles which connected with each other (Fig. S2†). Static contact angles of water drops (15 μL) on the pressed pellet of the PPy powder was measured to be 127 ± 4°, proving the powder's hydrophobicity. The PPy powder coating on the pins rendered them hydrophobic and non-wetting to water (Fig. 2c and d).

**Fig. 3:**
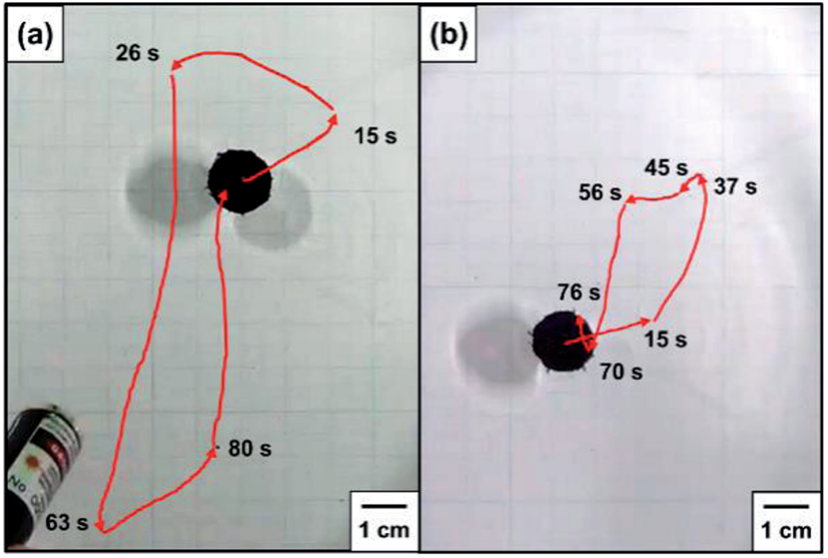
Stereomicrograph images illustrating locomotions of pins placed on the planar air–water interface with the pin point protruding into (a) air and (b) water phases.

**Fig. 4:**
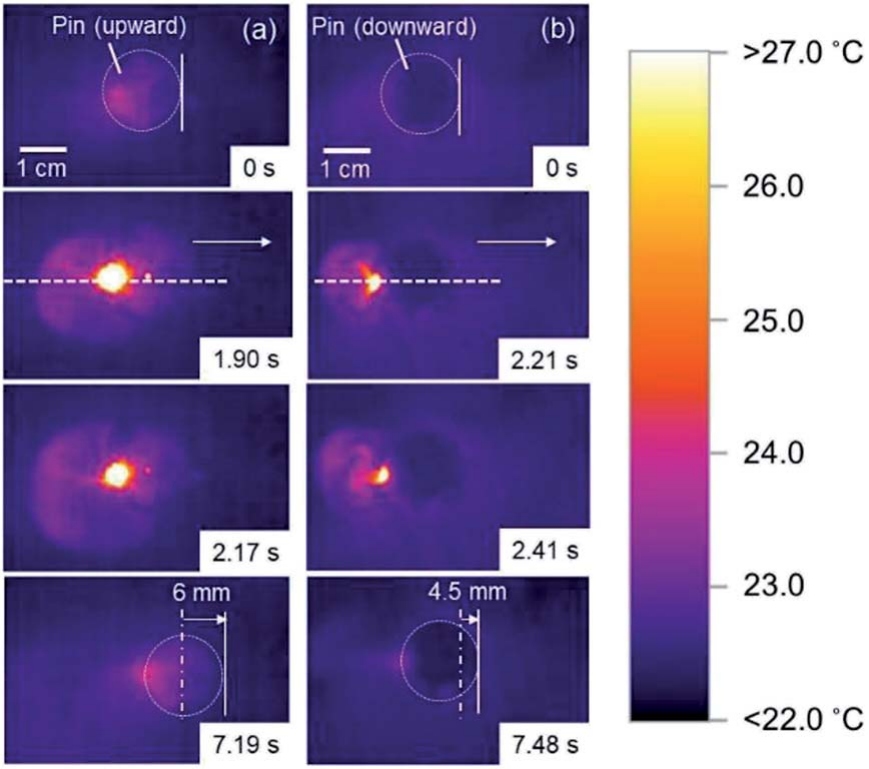
Snapshots of the typical light-driven locomotion of pins placed on the planar air–water interface with the pin point protruding into (a) air and (b) water phases observed by thermography. At *t* = 0, the pin was in thermal equilibrium. NIR irradiation caused a strong heating and locomotion of the pin. At *t* = 7.19 s in (a) and 7.48 s in (b), the locomotion distances are shown.

**Fig. 5:**
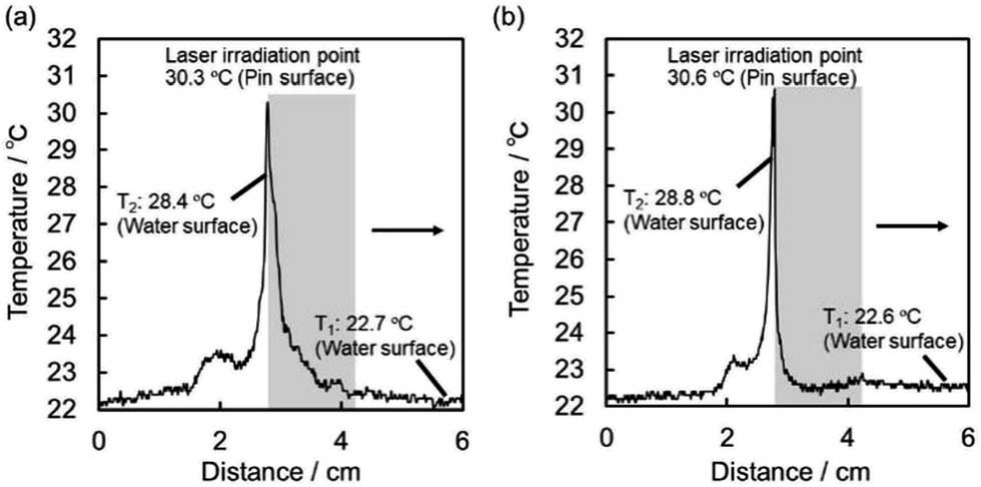
Temperature profiles obtained from the thermographs shown in Fig. 4(a and b), following the white dotted lines in the direction of the arrows at *t* = 1.90 and 2.21 s for pins placed on the planar air–water interface with the pin point protruding into (a) air and (b) water phases, respectively. The shaded areas indicate the pin existing places.

The black-colored pin placed on the planar air–water interface prepared in Petri dish, remained intact for more than 48 h, independent of direction of the pin (upward and downward). In order to move the pins on the planar air–water interface on demand, the three phase contact line of the pin head, air and water was manually irradiated by the NIR laser. The pins smoothly moved forward on the air–water interface away from the point of NIR irradiation. In Fig. 3a, the pin was placed with the pin point upward (a point protruding into air phase) in a Petri dish filled with water. The pin was irradiated and its motion was traced (highlighted using red arrows which indicate the direction, see also ESI Movie 1†). With one NIR irradiation shot, the pin gains force to move and travels on the air–water interface for a few tens seconds. When the movement stopped, another NIR irradiation shot was given to the pin. The locomotion directions can be easily controlled by the NIR irradiation directions. The average traveling distance per one shot was 5.3 ± 2.9 cm and the deviation should be due to difference of shot point on the pin head. The heat distribution at planar air–water interface upon irradiation of the pin was studied by thermography, in order to understand the motion process in detail. Typical thermography snapshots were shown for the light-driven locomotion of the pin placed with the pin point upward at the air–water interface in Fig. 4a. At *t* = 0 s, the pin is in thermal equilibrium with the surrounding planar water surface. Upon the NIR laser irradiation of the pin (snapshots at *t* = 1.90 and *t* = 2.17 s), the center of irradiation became the hottest spot, which immediately caused anisotropic heat flow. For analysis of the heat flow, temperature *vs.* position profile was recorded along the path of locomotion (snapshot *t* = 1.90 s). Fig. 5a suggests that the temperature of the NIR laser-irradiated point of the pin was >30 °C and a heat tail could be observed. In contrast to the bulk water (22.7 °C), water near the pin had a temperature of approximately 28 °C. This temperature difference leads to a surface tension difference Δ*γ* of approximately 0.96 mN m^−1^ in front and in the rear of the pin. This surface tension difference works as a driving force for the locomotion of the pin.

**Fig. 6:**
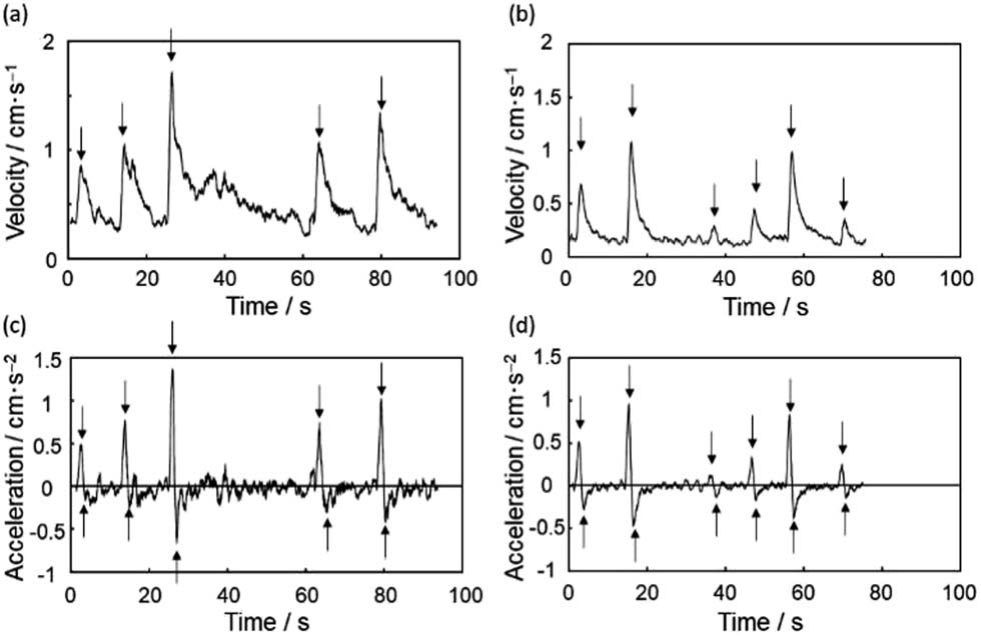
The motion of upward pin is shown with (a) velocity and (c) acceleration. The NIR is irradiated 5 times around arrows. The motion of downward pin is shown with (b) velocity and (d) acceleration. The NIR is irradiated 6 times around arrows.

The velocity and acceleration of the pin placed with the pin point upward are shown in Fig. 6a and c with 5 separate NIR irradiations as indicated by arrows. The velocity rapidly rises by each irradiation and decays exponentially. The average of five local maxima in velocities indicated by arrows (〈*v*_max_〉) is 1.20 cm s^−1^. The acceleration is proportional to forces acting on the pin. Its profile demonstrates that the induced force is pulse-like composed of positive and negative parts. The positive one results from NIR irradiation, and the average of five local maxima in accelerations indicated by arrows (〈*a*_max_〉) is 0.881 cm s^−2^. On the other hand, the negative one is caused by fluid resistance force, and the average of five local minima in accelerations indicated by arrows (〈*a*_min_〉) is −0.365 cm s^−2^.

The locomotion of pins on the air–water interface could be also induced by NIR laser irradiation when the pin was placed with the pin point downward (a point protruding into water phase) (Fig. 3b and ESI Movie 2†) and its direction can be changed by the irradiation directions again. Thermography snapshots of NIR laser-driven locomotion of the pin indicate anisotropic heat flow on the water surface again (Fig. 4b). Considering that the temperature difference between bulk water surface (22.6 °C) and water surface near the pin (28.8 °C) (Fig. 5b), the difference in surface tension observed for the pin point downward system was determined to be 1.04 mN m^−1^, which is similar to that observed for the pin point upward system. The average traveling distance per one shot was 2.0 ± 1.4 cm, which were 2.65 times shorter than those for the pin point upward system.

The velocity and acceleration of the pin placed with the pin point downward are shown in Fig. 6b and d with 6 separate NIR irradiations as indicated by arrows. The profiles are similar to those of upward case. The average of local maxima in velocities is 0.645 cm s^−1^ (〈*v*_max_〉), and the averages of local maxima and minima in accelerations indicated by arrows are 0.501 cm s^−2^ (〈*a*_max_〉) and −0.27 cm s^−2^ (〈*a*_min_〉), respectively. The downward/upward ratios for 〈*v*_max_〉 and 〈*a*_max_〉 are determined to be 0.538 and 0.569, respectively. The traveling distance per one shot, velocity and acceleration were appreciably attenuated comparing to those observed in upward system, which should be due to the fluid resistance from the pin point in bulk water phase.

The drag force *F*_drag_ caused by fluid resistance can be expressed using an equation, as shown below.^29^1
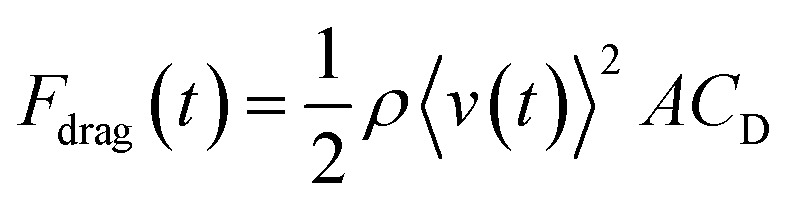
Here, the *A* is a projected area for a moving object in fluid. The *ρ* and *C*_D_ are density of the fluids and fluid resistance coefficients. In general, *C*_D_ depends on Reynolds number Re that can be calculated by *ρ*〈*v*(*t*)〉*L*/*μ*, where *L* and *μ* represent the characteristic linear dimension and viscosity of fluid, respectively. Because both the viscosity and the density of water are almost 1000 times greater than those of air,^30^ the Re and the resultant *C*_D_ values for water and air are not much different. Because of the similar *C*_D_ values, the *F*_drag_ in eqn (1) could be thought to be proportional to the fluid density, and *F*_drag_(*t*) of an object in air can be ignored compared to that in water. Due to the pin weight, meniscus is formed at air–water interface, which keeps the pin on water and a small part of pin head is soaked in water. Based on this situation, the fluid resistance should be mainly caused by the pin point protruding in water (downward pin) and the pin head soaked in water (upward and downward pins). These projection areas in water were used as *A* values. The interface between pin head and water may also affect the fluid resistance due to the viscous drag (see below).

**Fig. 7:**
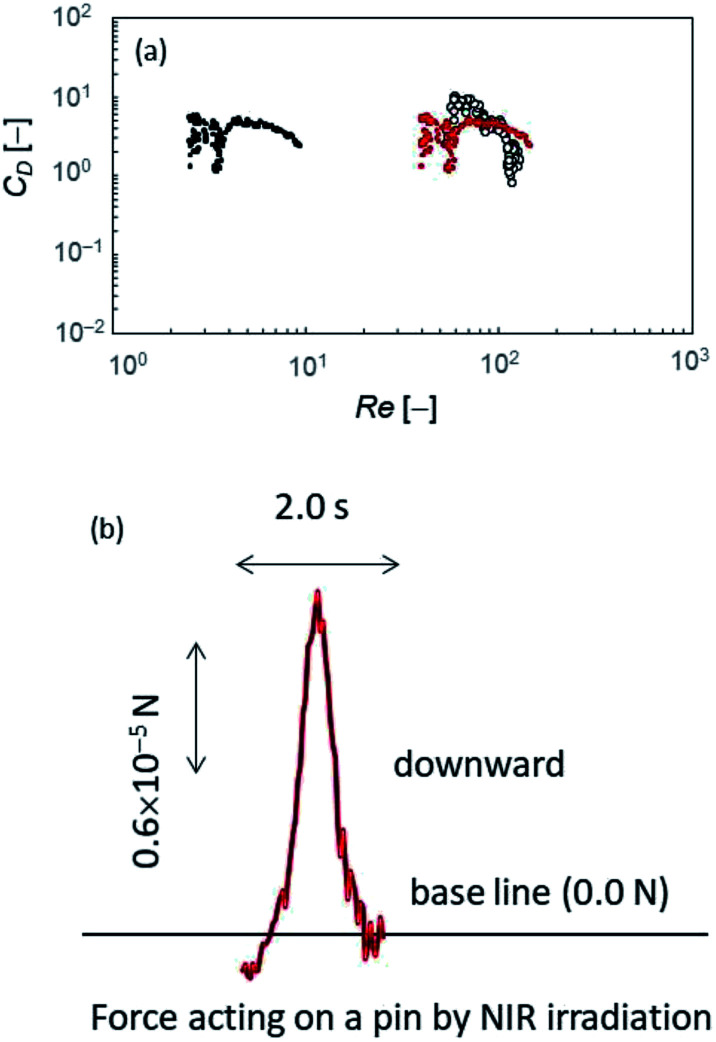
(a) The fluid resistance coefficients and the driving force by NIR irradiation evaluated with the resistance coefficient. *C*_D_ is shown with Re. The solid dots are the values of downward pin. The larger and smaller Reynolds number are calculated with *L* = 14.3 mm and 0.9 mm, respectively. The open circle is the values of upward pin with *L* = 14.3 mm. (b) The driving force generated by NIR irradiation (*F*_NIR_(*t*)) evaluated with an average value of *C*_D_.

After the NIR irradiation was ceased and a little time had passed for the temperature gradient around the push pin to disappear, the fluid resistance was the only force acting on the object, and *F*_drag_ can be calculated using *m*〈*a*(*t*)〉. For the calculation of Re, the diameter of pin head was used as *L* value for upward pin, as the pin point is in the air. On the other hand, two characteristic *L* values (*i.e.*, the diameters of pin head and pin point) were used for downward pin. Depending on the *L* value, different Re values could be obtained: larger *L* value (the pin head diameter) results in larger Re values. *C*_D_ can be calculated as a function of Re from eqn (1): two different Re values can be plotted on the same *C*_D_ values for the downward pin. This calculation is applied for the 1st laser-induced locomotion for upward pin (Fig. 6a and c) and the 2nd one for downward pin (Fig. 6b and d), because they are almost pure translation without rotation on air–water interface. Fig. 7a shows that the *C*_D_ is almost constant independently of Re for the downward pin. This suggests that the fluid resistance is mainly caused by the inertia effect of pin point in water. On the other hand, the *C*_D_ decreases with an increase in Re, for the upward pin. This suggests that the fluid resistance is caused by viscous drag. (Note that in the case of a small value of Re, the *C*_D_ should be inversely proportional to Re.) This viscous drag may arise from a pin head soaked in water and from the interface between pin head and water. The *C*_D_ in Fig. 7a is calculated with the *A* value for the pin head. Thus, the absolute value may contain uncertainty to a large extent. Nevertheless, we may consider that the fluid resistance is mainly caused by viscous drag because the *A* value does not affect the dependency of *C*_D_ on Re.

As discussed above, the *C*_D_ for downward pin obtained in Fig. 7a is more reliable compared with that for upward pin. Thus, we used the experimental results and *C*_D_ value for downward pin to obtain the driving force by the laser irradiation *F*_NIR_(*t*). The *F*_NIR_(*t*) for upward pin must be in the same order of magnitude, because *F*_NIR_(*t*) is generated by the same mechanism independently of the pin setup. We used a constant value of *C*_D_ to calculate the drag force (*F*_drag_(*t*)) during the period when the NIR laser was irradiated. (*C*_D_ = 5.5 that is an average of *C*_D_ shown in Fig. 7a). The driving force generated by the NIR irradiation, *F*_NIR_(*t*), was calculated from *F*(*t*) − *F*_drag_(*t*) (Fig. 7b). The force generated by NIR irradiation is 10^−5^ N in the order of magnitude. Since the diameter of pin head where surface tension difference operates is 14.3 mm, this force corresponds to 10^−1^ to 10^0^ mN m^−1^ in the order of magnitude. Temperature increase induced by NIR irradiation is approximately 6 °C (Fig. 5), leading to 1 mN m^−1^ in the order of magnitude of surface tension change.^31^ This value agrees with the above-estimated value in the order of magnitude. The object is driven by surface tension change by NIR irradiation, and the motion is dragged depending on its geometry: the downward pin has a pin point protruding into water, which increases the drag force due to the inertia effect. This effect reduces the velocity.

## Conclusions

In summary, we succeeded in moving centimeter-sized pin on the planar air–water interface by simple light irradiation. It was described that the existing place of the protruding part of the pin is one of crucial parameters which controls the light-driven locomotion of the pin. Velocity and acceleration were smaller and traveling distance was shorter when the pin was placed with a point protruding into water phase due to larger friction of water, comparing when the pin was placed with a point protruding into air phase. Wide varieties and simplicity of the coating method of small objects with various shapes by photothermal materials will enable synergistic experimental and theoretical investigations toward the understanding and utilization of light-controlled powering, manipulating and delivery system.

## Conflicts of interest

There are no conflicts to declare.

## Supplementary Material

RA-009-C9RA01417A-s001

RA-009-C9RA01417A-s002

RA-009-C9RA01417A-s003
