# Supplementary material for: Light-driven locomotion of a centimeter-sized object at the air–water interface: effect of fluid resistance
Source: RSC Adv. 2019 Mar 13;9(15):8333–9. doi: 10.1039/c9ra01417a (PMC9061706; doi:10.1039/c9ra01417a)
Supplement: RA-009-C9RA01417A-s001 [file RA-009-C9RA01417A-s001.pdf]

Electronic Supplementary Material (ESI) for RSC Advances.
This journal is © The Royal Society of Chemistry 2019

Electronic Supplementary Infomaton

Light-driven locomotion of centimeter-sized object on air-water interface:

Effect of fluid resistance

Hisato Kawashima[a], Akihisa Shioi[b]*, Richard J. Archer[c], Stephen J. Ebbens[c]

Yoshinobu Nakamura[d,e], Syuji Fujii[d,e]*

[a] *Division of Applied Chemistry, Graduate School of Engineering, Osaka Institute of Technology, 5-16-1 Omiya, Asahi-ku, Osaka, 535-8585, Japan*

[b] *Department of Chemical Engineering and Materials Science Doshisha University, Kyoto 610-0321, Japan*

[c] *Department of Chemical and Biological Engineering, The University of Sheffield, Mappin Street, Sheffield S1 3JD, UK*

[d] *Department of Applied Chemistry, Faculty of Engineering, Osaka Institute of Technology, 5-16-1 Omiya, Asahi-ku, Osaka, 535-8585, Japan*

[e] *Nanomaterials Microdevices Research Center, Osaka Institute of Technology, 5-16-1 Omiya, Asahi-ku, Osaka, 535-8585, Japan*

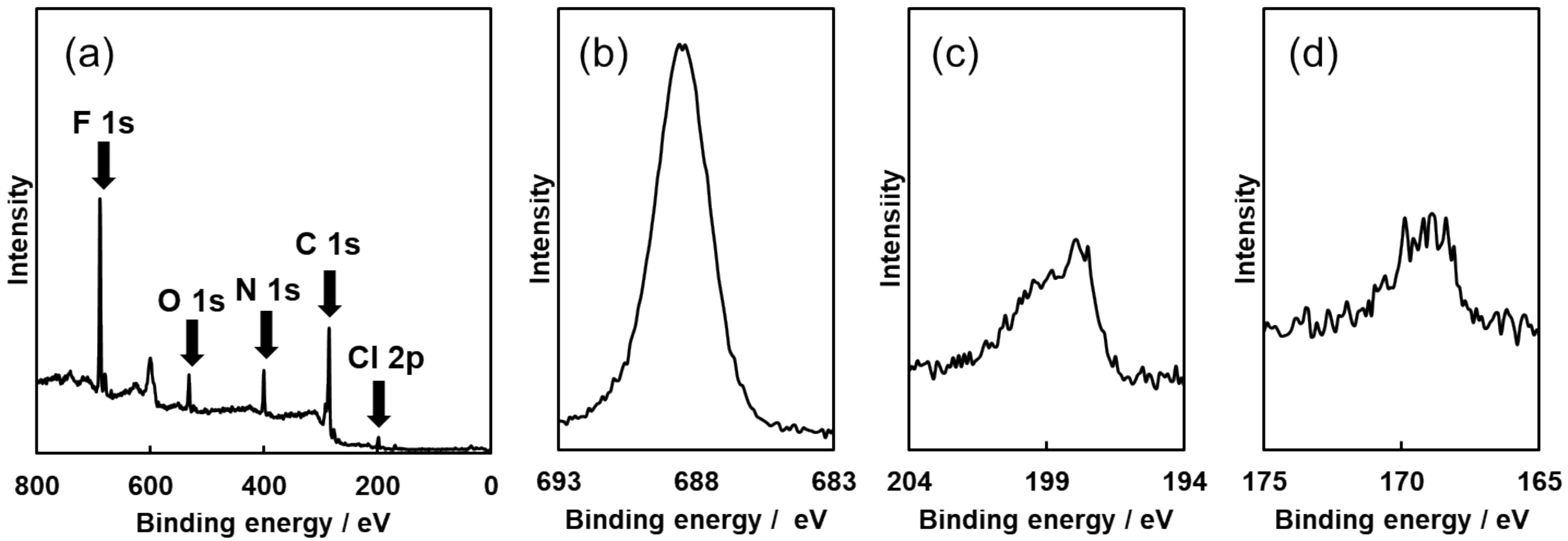

(e)

	Atom/%					
	C	O	N	S	Cl	F
PPy-C_8F[a)]	61.44	5.41	10.10	0.88	1.44	20.73

[a)]Py/C_8F=3/0.4

Figure S1. X-ray photoelectron spectroscopy (a) survey spectrum, (b) F 1s spectrum, (c) Cl 2p spectrum and (d) S 2p spectrum of PPy-C8F powder. (e) Surface atomic compositions of the PPy-C8F powder.

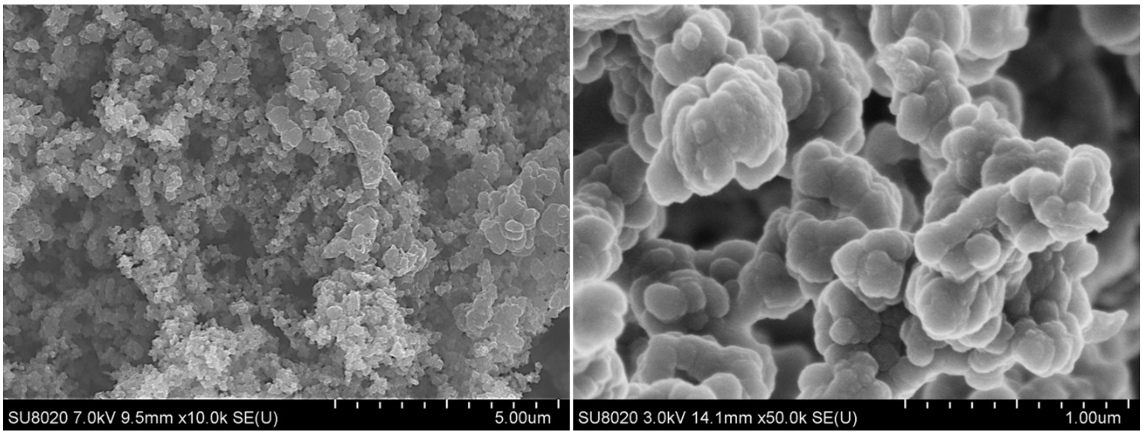

Figure S2. SEM images of PPy-C8F powder (Py/C8F=3/0.4).
